# Oral β-Lactams, Fluoroquinolones, or Trimethoprim-Sulfamethoxazole for Definitive Treatment of Uncomplicated *Escherichia coli* or *Klebsiella* Species Bacteremia From a Urinary Tract Source

**DOI:** 10.1093/ofid/ofad657

**Published:** 2023-12-27

**Authors:** Sameer Alzaidi, John J Veillette, Stephanie S May, Jared Olson, Katarina Jackson, C Dustin Waters, Allison M Butler, Mary A Hutton, Whitney R Buckel, Brandon J Webb

**Affiliations:** Department of Pharmacy, Intermountain Health, Taylorsville, Utah, USA; Infectious Diseases Telehealth Service, Intermountain Health, Murray, Utah, USA; Department of Pharmacy, Intermountain Medical Center, Murray, Utah, USA; Infectious Diseases Telehealth Service, Intermountain Health, Murray, Utah, USA; Department of Pharmacy, Intermountain Medical Center, Murray, Utah, USA; Department of Pharmacy, Primary Children's Hospital, Salt Lake City, Utah, USA; Division of Infectious Diseases, Department of Pediatrics, University of Utah, Salt Lake City, Utah, USA; Department of Pharmacy, Intermountain Medical Center, Murray, Utah, USA; Department of Pharmacy, McKay-Dee Hospital, Ogden, Utah, USA; Statistical Data Center, Intermountain Health, Murray, Utah, USA; Department of Pharmacy, Utah Valley Hospital, Provo, Utah, USA; Department of Pharmacy, Intermountain Health, Taylorsville, Utah, USA; Division of Clinical Epidemiology and Infectious Diseases, Intermountain Medical Center, Murray, Utah, USA

**Keywords:** antimicrobial stewardship, β-lactams, gram-negative bacteremia, real-world evidence, urinary tract infection

## Abstract

**Background:**

Fluoroquinolones (FQs) are effective for oral step-down therapy for gram-negative bloodstream infections but are associated with unfavorable toxic effects. Robust data are lacking for trimethoprim-sulfamethoxazole (TMP-SMX) and high-bioavailability β-lactams (HBBLs).

**Methods:**

In this multicenter observational cohort study, we simulated a 3-arm registry trial using causal inference methods to compare the effectiveness of FQs, TMP-SMX, or HBBLs for gram-negative bloodstream infections oral step-down therapy. The study included adults treated between January 2016 and December 2022 for uncomplicated *Escherichia coli* or *Klebsiella* species bacteremia of urinary tract origin who were who were transitioned to an oral regimen after ≤4 days of effective intravenous antibiotics. Propensity weighting was used to balance characteristics between groups. 60-day recurrence was compared using a multinomial Cox proportional hazards model with probability of treatment weighting.

**Results:**

Of 2571 patients screened, 648 (25%) were included. Their median age (interquartile range) was 67 (45–78) years, and only 103 (16%) were male. Characteristics were well balanced between groups. Compared with FQs, TMP-SMX had similar effectiveness (adjusted hazard ratio, 0.91 [95% confidence interval, .30–2.78]), and HBBLs had a higher risk of recurrence (2.19 [.95–5.01]), although this difference was not statistically significant. Most HBBLs (70%) were not optimally dosed for bacteremia. A total antibiotic duration ≤8 days was associated with a higher recurrence rate in select patients with risk factors for failure.

**Conclusions:**

FQs and TMP-SMX had similar effectiveness in this real-world data set. HBBLs were associated with higher recurrence rates but suboptimal dosing may have contributed. Further studies are needed to define optimal BL dosing and duration to mitigate treatment failures.

## Graphical Abstract

**Graphical Abstract:**
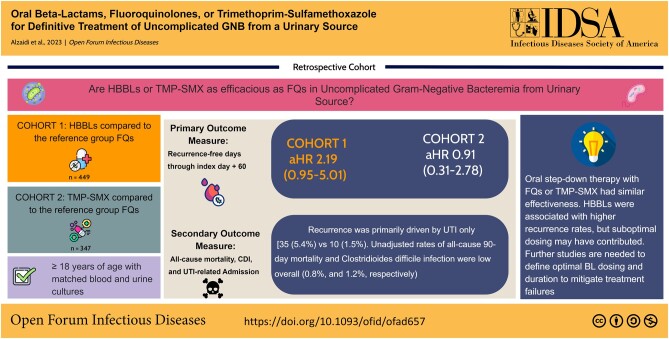
https://tidbitapp.io/tidbits/oral-beta-lactams-fluoroquinolones-or-trimethoprim-sulfamethoxazole-for-definitive-treatment-of-uncomplicated-escherichia-coli-or-klebsiella-species-bacteremia-from-a-urinary-source

## Teaser

Oral stepdown with fluoroquinolones or trimethoprim-sulfamethoxazole had similar effectiveness for uncomplicated GN-BSI from a urinary source. High-bioavailability β-lactam (HBBL) stepdown was associated with higher recurrence but was limited by suboptimal dosing. How best to mitigate HBBL recurrences requires further study.

Gram-negative bloodstream infections (GN-BSIs) originating from a urinary tract source are commonly encountered in hospitalized patients. Step-down oral antibiotic therapy is increasingly prescribed as a safer, less costly, and more convenient alternative to outpatient intravenous antibiotics [1–4]. However, available evidence remains incomplete regarding the optimal oral step-down agent and duration. Efficacy data are most robust for fluoroquinolones (FQs) [5, 6], but these agents have important safety concerns including high risk for *Clostridioides difficile* infection [7, 8]. Trimethoprim-sulfamethoxazole (TMP-SMX) also has recognized toxic effects, and data regarding its effectiveness for GN-BSI are limited to small retrospective studies, which often combined TMP-SMX with FQs in their analyses [9–12].

There is growing interest in oral β-lactam (BL) antibiotics, particularly high-bioavailability BLs (HBBLs), owing to fewer adverse effects and preserved susceptibility rates among Enterobacterales. However, evidence regarding BL effectiveness for oral step-down therapy in GN-BSI remains inconclusive. Several retrospective studies have found no difference between BLs and FQs [13–15], whereas other studies, including a meta-analysis, have found BLs to be associated with a higher rate of recurrent infection [12, 16, 17]. These data have many limitations, including various retrospective study designs, heterogeneous patient populations, and variable definitions and time frames for evaluating treatment failure. In the absence of randomized controlled trials, a comparative effectiveness study using causal inference methods is needed to add clarity to the available evidence. Herein, we present real-world data from a large, observational, multicenter cohort study using a target trial emulation to compare effectiveness of oral step-down HBBLs, FQs, and TMP-SMX for uncomplicated GN-BSIs from a urinary tract source.

## METHODS

### Patients and Data Collection

The study took place in the Intermountain Health integrated network of 23 hospitals and emergency departments (EDs), 38 urgent care facilities, and 300 primary care clinics in Utah and Idaho, serving more than 1.5 million patients each year. Using the Intermountain enterprise data warehouse, we identified a screening population comprising unique patients ≥18 years of age who had matching, simultaneously collected, positive blood and urine cultures for *Escherichia coli*, *Klebsiella pneumoniae*, or *Klebsiella oxytoca* during an ED encounter or hospital admission between January 2016 and December 2022.

Data related to hospital course, demographics, laboratory values, and microbiology were extracted electronically from the enterprise data warehouse, whereas comorbid conditions, imaging, severity of illness, antibiotic treatment, recurrent infection, readmissions, and mortality data were abstracted manually from the electronic medical record (EMR) by trained record reviewers using a standardized data collection tool (see Supplementary Materials). Data were collected for all patients through 90 days after hospital discharge. The study met all STROBE requirements for observational studies [18] and ISPOR criteria for comparative effectiveness [19]. The study was approved by the Intermountain Healthcare Institutional Review Board and was granted a waiver of patient consent owing to its design and less-than-minimal risk to subjects.

### Exclusion Criteria

From the initial screening cohort, we excluded the following patients: those with concomitant infections (besides GN-BSIs/urinary tract infections [UTIs]) during the index visit, polymicrobial cultures, hospital-onset bacteremia, non–urinary tract source of bacteremia (eg, prostatitis, epididymo-orchitis), or complicated UTIs (defined as benign prostatic hypertrophy, bladder or prostate cancer, urinary obstruction/retention, hydronephrosis, kidney or bladder stones, neurogenic bladder, chronic urinary incontinence, indwelling or intermittent urinary catheterization, renal abscess, or altered urologic anatomy—eg, stent, tube, sling, urostomy, cystocele, fistula, or stricture). We also excluded patients who died in the hospital, were discharged to hospice care, transferred to a non-Intermountain facility, were lost to follow-up after discharge (with no notes or visits in the record thereafter), were pregnant, or were immunocompromised (human immunodeficiency virus/AIDS with CD4 cell count <200/µL, neutropenia with an absolute neutrophil count <500/µL, or receiving any of the following medications at the time of admission: antirejection medications after transplantation, chemotherapy, tumor necrosis factor α inhibitors, disease-modifying antirheumatic drugs, or maintenance steroids with equivalent prednisone dose ≥20 mg). Patients were also excluded if they had a prolonged hospital admission (>14 days), did not receive an effective intravenous antibiotic within 24 hours of the index blood culture, received >4 days of intravenous antibiotic therapy, were discharged on an intravenous antibiotic, received multiple oral antibiotics, received an oral antibiotic to which the blood or urine isolate was not susceptible, or had incomplete EMR data regarding antibiotic treatment.

### Microbiology Procedures

Blood culture susceptibilities were performed on either BD Phoenix (BD Diagnostic Systems) or MicroScan Walkaway (Beckman Coulter) panels depending on the processing facility, whereas all urine cultures were processed on MicroScan Walkaway panels. Granular minimum inhibitory concentration (MIC) data were lacking for many isolates reported only as “susceptible” in our EMR, and we often had to infer susceptibility for oral antibiotics (eg, cephalexin and amoxicillin) based on surrogate intravenous antibiotics reported on the panel (eg, cefazolin and ampicillin, respectively). Because of this limitation, we preplanned an analysis limited to isolates confirmed to be susceptible by current Clinical and Laboratory Standards Institute (CLSI) breakpoints (see Sensitivity Analyses section).

### Enrollment Window and Index Day Zero

The index day zero for the simulated trial was defined as the date that the original positive blood cultures were obtained. An enrollment window was defined as index day +1 through day +4 to reflect real-world variation in the time to culture positivity and time to clinical stability. Patients were permitted to transition from intravenous to oral antibiotics at any time during the enrollment window per the treating physician's discretion, provided that they were transitioned to definitive oral step-down therapy by index day +4.

### Comparator Groups

Patients were classified at the time of enrollment into 1 of 3 comparator groups based on the definitive oral antibiotic they received: FQs (levofloxacin and ciprofloxacin); TMP-SMX; or HBBLs (amoxicillin, amoxicillin-clavulanate, and cephalexin). For sensitivity analyses, we also evaluated a fourth group of low-bioavailability BLs (LBBLs; cefdinir and cefuroxime). FQs were used as the reference group in all comparisons. Oral antibiotic dosing and duration was per prescriber choice in this real-world study. However, we prespecified a descriptive analysis of patients who received bacteremia dosing per Delphi expert consensus recommendations from Heil et al [20] (with appropriate adjustments for renal impairment): ciprofloxacin, 750 mg orally every 12 hours; levofloxacin, 750 mg orally every 24 hours; TMP-SMX, 5 mg/kg orally every 12 hours (eg, approximately 2 double-strength tablets every 12 hours for a 70-kg patient); amoxicillin, 1000 mg orally every 8 hour; amoxicillin–clavulanic acid, 875–1000 mg orally every 8 hours; and cephalexin, 1000 mg orally every 6 hours [20].

### Outcomes and Primary Analysis

The primary outcome was recurrence-free days through index day +60. Recurrence was defined as positive blood or urine culture for the same organism (regardless of susceptibility results). If only the urine culture was positive (ie, no evidence of recurrent bacteremia), manual review of the EMR had to indicate that the patient had a symptomatic UTI diagnosed and treated with antibiotics for the case to count as a recurrence. Otherwise, positive urine cultures without documented symptoms and treatment were classified as asymptomatic bacteriuria and were not counted toward the primary outcome. Secondary outcomes included all-cause mortality, *C difficile* infection, and UTI-related readmission. All ED visits or hospital readmissions were manually reviewed to determine whether they were UTI related, which was defined as either an adverse event to the initial antibiotic treatment or ongoing, worsening, recurrent UTI as the reason for readmission. To ensure consistency, adjudication of all patients with a primary or secondary outcome was performed by the same infectious diseases pharmacist (J. J. V.).

### Statistical Analysis

To address the principle of exchangeability in the trial emulation, we used a 3-comparator multinomial regression model to estimate propensity weights for choosing one antibiotic group over the others (R, twang package) [21–24]. The propensity model was fitted using covariates identified from recent GN-BSI propensity-adjusted studies [1, 25] and causal diagrams, including age, sex, chronic kidney disease, Charlson comorbidity index, Pitt bacteremia score, allergy or resistance to FQs, TMP-SMX, or BLs, and hospital size <200 beds (owing to observed variation in prescribing patterns between large and small hospitals in our health system). We selected an optimally balanced model for the primary analysis based on absolute standardized mean differences, minimum *P* values, and effective sample sizes (Supplementary Table 1). We also assessed covariate overlap via propensity score box plots, which, together with the balance table, supported causal estimation (Supplementary Figure 1). We then compared recurrence risk between groups using a propensity score–weighted multinomial Cox proportional hazards model. The model was censored at the time of death, administration of antibiotics during a subsequent ED/hospital admission (for indications other than GN-BSI/UTI), or last known follow-up within 90 days (rather than prespecifying separate per-protocol and intention-to-treat analyses). The duration of intravenous antibiotics (in days) was included as a time-dependent covariate in the model because the enrollment window allowed for a several-day range of intravenous antibiotic treatment before the switch to oral antibiotics (hereafter, “oral switch”).

Regarding antibiotic treatment duration, in a slight divergence from strict trial emulation methods (where duration subgroups would ideally be stratified at enrollment), we chose to optimize power and recognize uncertainty in the existing evidence [25–27] by including total antibiotic duration as a continuous, time-dependent variable in the primary analysis. We also conducted a prespecified analysis to explore the association between total duration and recurrence. To determine an optimal cutoff point for dichotomizing duration, we used a receiver operating characteristic curve to plot 60-day recurrence versus antibiotic duration and calculated the Youden index [28], which optimized sensitivity and specificity for all antibiotics. We then refit the primary Cox models, using the duration cutoff point as an interaction term, to estimate the effect of each oral antibiotic on recurrence stratified by short versus long duration. Results were displayed by creating cumulative incidence plots.

### Sensitivity Analyses

Several sensitivity analyses were planned a priori. First, we evaluated the primary analysis for recurrence at index day +30 and index day +90. Second, we expanded the BL group in the primary analysis to include all BLs (LBBLs and HBBLs together). Third, we limited the BL group in the primary analysis to only those patients with HBBLs whose blood and urine isolates were confirmed to be susceptible at current CLSI breakpoints (cefazolin MIC ≤2 mg/L if they received cephalexin, ampicillin MIC ≤8 mg/L if they received amoxicillin, or amoxicillin-clavulanate MIC ≤8/4 mg/L if they received amoxicillin-clavulanate) [29]. Fourth, we conducted a variation of the primary analysis using expanded inclusion criteria allowing up to 7 days (instead of 4 days) of intravenous antibiotics before the oral switch. Finally, we fitted a new binomial Cox model to compare LBBLs and HBBLs.

## RESULTS

**Figure 1.**
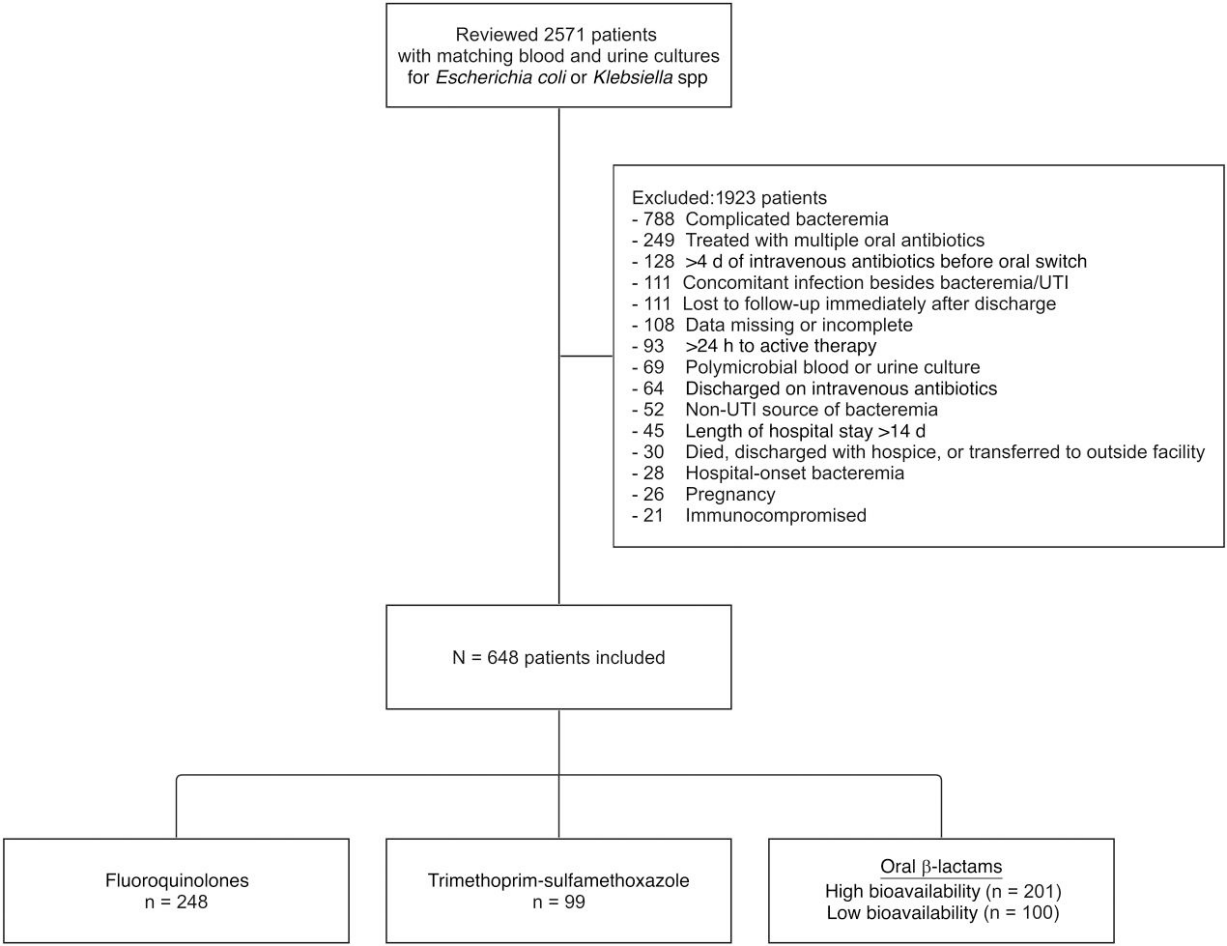
Patient inclusion/exclusion. Abbreviation: UTI, urinary tract infection.

Of 2571 patients reviewed during the 6-year study period, 648 (25%) met inclusion criteria (Figure 1), including 248 patients in the FQ group, 99 in the TMP-SMX group, and 201 in the HBBL group. Most patients (n = 512 [79%]) were treated in the hospital, and 63 (10%) required intensive care (Table 1). Patients were predominantly female (545 [84%]) and >65 years of age (n = 337 [52%]). Demographics, comorbid conditions, severity of illness, microbiology, and antibiotic treatment characteristics were similar across the oral step-down antibiotic groups. Nearly all patients (n = 640 [99%]( achieved clinical stability (ie, were afebrile and hemodynamically stable) within the first 3 days. Most patients (n = 635 [98%]) were initially treated with an intravenous BL before oral step-down. The median time to oral switch (interquartile range) was 3 (2–4) days. Oral antibiotic prescribing patterns varied by care venue: ED patients were most likely to receive a BL (60 of 136 [44%]), hospitalized patients were most likely to receive an FQ (213 of 512 [42%]), and patients transitioned to TMP-SMX more frequently received care at smaller hospitals (58 of 99 [59%]). The median total duration of antibiotics (interquartile range) was 11 (10–14) days. Patients treated with an FQ were more likely to receive consensus-recommended dosing than those treated with TMP-SMX (59% vs 3%, respectively; *P* < .001 by Fisher exact test) or HBBLs (59% vs 30%; *P* < .001) (Table 1).

**Table 1.** Demographics, Comorbid Conditions, and Antibiotic Treatment

Variable	Patients by Antibiotic Treatment, No. (%)^a^				
	All Patients (N = 648)	FQs (n = 248)	TMP-SMX (n = 99)	HBBLs (n = 201)	LBBLs^b^ (n = 100)
Age, median (IQR), y	67 (46–78)	64 (45–76)	68 (49–80)	70 (47–79)	69 (51–81)
Female sex	545 (84)	209 (84)	75 (76)	178 (89)	83 (83)
Diabetes mellitus	266 (41)	102 (41)	47 (47)	89 (44)	28 (28)
Chronic kidney disease (stage II or higher)	157 (24)	53 (21)	26 (26)	51 (25)	27 (27)
Charlson comorbidity score, median (IQR)	4 (2–8)	4 (2–8)	4 (2–8)	5 (1–8)	5 (2–7)
History of kidney stones	22 (3)	10 (4)	2 (2)	9 (4)	1 (1)
>2 Positive urine cultures in past year	14 (2)	6 (2)	2 (2)	3 (1)	3 (3)
Allergy to FQ, TMP-SMX, or β-lactam	27 (4)	15 (6)	0 (0)	5 (2)	7 (7)
Received care at small hospital (<200 beds)	276 (43)	89 (36)	58 (59)	82 (41)	47 (47)
Length of hospital stay, median (IQR), d	2.5 (0.2–3.0)	2.6 (1.4–3.1)	2.8 (1.9–3.3)	2.0 (0.2–3.0)	2.1 (0.2–2.9)
Highest level of care					
Emergency department	136 (21)	35 (14)	13 (13)	60 (30)	28 (28)
Inpatient	512 (79)	213 (86)	86 (87)	141 (70)	72 (72)
Severity of illness					
Pitt bacteremia score, median (IQR)	1 (1–3)	1 (1–3)	1 (1–3)	1 (1–2)	1 (1–2)
Admitted to ICU	63 (10)	28 (11)	6 (6)	15 (7)	14 (14)
Received vasopressors	19 (3)	10 (4)	1 (1)	4 (2)	4 (4)
Achieved clinical stability within 3 d	640 (99)	244 (98)	98 (99)	199 (99)	99 (99)
Microbiology					
*Escherichia coli*	602 (93)	231 (93)	88 (89)	191 (95)	92 (92)
*Klebsiella* species	46 (7)	17 (7)	11 (11)	10 (5)	8 (8)
FQ resistant	48 (7)	0 (0)	18 (18)	22 (11)	8 (8)
TMP-SMX resistant	116 (18)	52 (21)	0 (0)	43 (21)	21 (21)
Cefazolin resistant	52 (8)	25 (10)	19 (19)	0 (0)	8 (8)
ESBL-producing isolate	8 (1)	3 (1)	5 (5)	0 (0)	0 (0)
Empiric intravenous antibiotic					
β-Lactams	635 (98)	238 (96)	99 (100)	199 (99)	99 (99)
FQs	13 (2)	10 (4)	0 (0)	2 (1)	1 (1)
Time to active therapy, median (IQR), h	1.3 (0.6–2.0)	1.2 (0.6–1.9)	1.4 (0.6–2.1)	1.3 (0.7–2.1)	1.4 (0.7–2.1)
Duration of active therapy, median (IQR), d	3 (2–4)	3 (2–4)	3 (3–4)	3 (2–4)	3 (1–4)
Definitive oral antibiotic					
Days of active oral therapy, median (IQR)	10 (7–10)	10 (7–11)	8 (7–10)	8 (7–10)	10 (7–10)
Received recommended dosing^c^	209 (32)	146 (59)	3 (3)	60 (30)	NA^d^
Total antibiotic duration, median (IQR), d	11 (10–14)	11 (10–14)	11 (10–14)	11 (9–13)	11 (10–14)

Abbreviations: ESBL, extended-spectrum β-lactamase; FQs, fluoroquinolones; HBBLs, high-bioavailability β-lactams; ICU, intensive care unit; IQR, interquartile range; LBBLs, low-bioavailability β-lactams; NA, not applicable; TMP-SMX, trimethoprim-sulfamethoxazole.

^a^Data represented No. (%) of patients unless otherwise specified.

^b^The LBBL group was included in sensitivity analyses but not in the primary analysis.

^c^Recommended oral antibiotic doses were based on consensus guidance from Heil et al [20].

^d^LBBLs are not routinely recommended for gram-negative bloodstream infections owing to pharmacokinetic concerns and lack of clinical data.

Crude 60-day recurrence occurred in 45 patients (6.9%) and was lowest in those receiving FQ step-down (n = 12 [4.8%]), largely owing to lower rates of recurrent bacteremia (n = 1 [0.4%]) relative to TMP-SMX and HBBLs (Table 2). Recurrence was primarily driven by UTI only (n = 35 [5.4%] vs 10 [1.5%] for recurrent bacteremia plus UTI). Unadjusted rates of all-cause 90-day mortality and *C difficile* infection were low overall (0.8%, and 1.2%, respectively) and did not differ significantly between groups (Table 2).

**Table 2.** Unadjusted Primary and Secondary Outcomes

Outcome	Patients by Antibiotic Treatment, No. (%)				
	All Patients (N = 648)	FQs (n = 248)	TMP-SMX (n = 99)	HBBLs (n = 201)	LBBLs (n = 100)
Primary outcome^a^					
60-d recurrence	45 (6.9)	12 (4.8)	8 (8.1)	16 (8.0)	9 (9.0)
Recurrent bacteremia + UTI	10 (1.5)	1 (0.4)	3 (3.0)	4 (2.0)	2 (2.0)
Recurrent UTI only	35 (5.4)	11 (4.4)	5 (5.1)	12 (6.0)	7 (7.0)
Secondary outcomes					
30-d Recurrence	22 (3.4)	7 (2.8)	3 (3.0)	9 (4.5)	3 (3.0)
90-d Recurrence	53 (8.2)	14 (5.6)	9 (9.1)	18 (9.0)	12 (12.0)
UTI-related readmission within 90 d^b^	56 (8.6)	21 (8.5)	9 (9.1)	16 (8.0)	10 (10.0)
*Clostridioides difficile* infection within 90 d	8 (1.2)	3 (1.2)	2 (2.0)	3 (1.5)	0 (0.0)
All-cause 90-d mortality	5 (0.8)	0 (0.0)	2 (2.0)	1 (0.5)	2 (2.0)

Abbreviations: FQs, fluoroquinolones; HBBLs, high-bioavailability β-lactam; LBBLs, low-bioavailability β-lactams; TMP-SMX, trimethoprim-sulfamethoxazole; UTI, urinary tract infection.

^a^Additional patients in each group who only had positive urine cultures by day +60 but no symptoms (ie, who did not meet criteria for recurrent UTI due to asymptomatic bacteriuria) are as follows: FQs, 0 patients; TMP-SMX, 0 patients; HBBLs, 6 patients; LBBLs, 0 patients.

^b^The following patients had 90-day UTI-related readmissions without meeting criteria for 90-day recurrence: FQ group, 12 patients (9 with new UTIs not growing index pathogen, 2 with ongoing/worsening symptoms due to index UTI, and 1 with an adverse event); TMP-SMX group, 5 patients (3 with new UTIs not growing index pathogen and 2 with adverse events); HBBL group, 4 patients (3 with new UTIs not growing index pathogen and 1 with ongoing/worsening symptoms); LBBL group, 3 patients (2 with new UTIs not growing index pathogen and 1 with ongoing/worsening symptoms).

**Figure 2.**
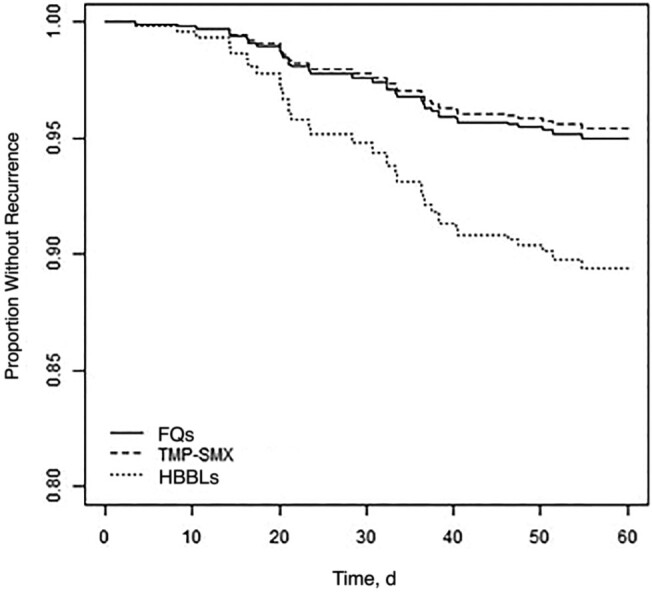
Recurrence-free days (through day +60) for uncomplicated gram-negative bloodstream infection based on oral step-down antibiotic. Abbreviations: FQs, fluoroquinolones; HBBLs, high-bioavailability β-lactams; TMP-SMX, trimethoprim-sulfamethoxazole. Data represent cumulative incidence curves generated from the propensity-weighted Cox proportional hazards models.

In the primary analysis, 60-day recurrence in the TMP-SMX group was similar to that in the FQ group (adjusted hazard ratio [aHR], 0.91 [95% confidence interval (CI), .30–2.78]). Patients treated with HBBL step-down had a higher risk of 60-day recurrence (2.19 [95% CI, .95–5.01]; *P* = .06), although this difference did not reach statistical significance in the primary model (Figure 2).

**Figure 3.**
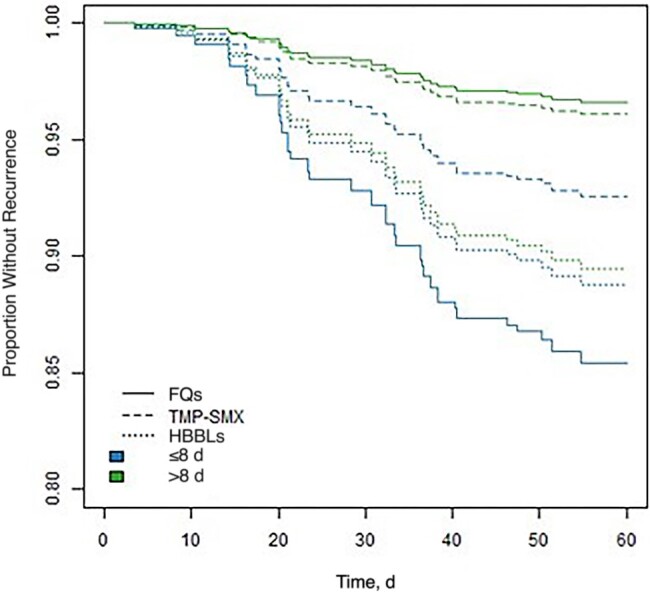
Recurrence-free days for gram-negative bloodstream infection based on oral step-down antibiotic and duration. Abbreviations: FQs, fluoroquinolones; HBBLs, high-bioavailability β-lactams; TMP-SMX, trimethoprim-sulfamethoxazole. Data represent cumulative incidence curves generated from the propensity-weighted Cox proportional hazards models.

Receiver operating characteristic curve analysis identified total antibiotic duration of 8 days as the best cutoff point in our data set for differentiating the effect of duration on 60-day recurrence, but the sensitivity and specificity of the curve were poor (Supplementary Figure 2). The modified primary analysis model stratified each oral antibiotic group by short (≤8 days) versus longer (>8 days) total duration. Compared with “short-course FQ” as the referent group, longer-course FQ therapy was associated with lower risk of recurrence (aHR, 0.22 [95% CI, .06–.82]; *P* = .02) (Figure 3). Recurrence risk for other treatment groups and durations, all relative to short-course FQ, were as follows: longer-course TMP-SMX (aHR, 0.25 [95% CI, .06–1.15]), short-course TMP-SMX (0.49 [.08–2.98]), short-course HBBL (0.76 [.2–2.93]), and longer-course HBBL (0.71 [.22–2.22]).

Results were robust to sensitivity analyses evaluating 30-day and 90-day recurrence and analyses using all BLs or low-cefazolin-MIC HBBLs as the third comparator group (Supplementary Figures 3–6). In the sensitivity analysis allowing up to 7 (instead of 4) days of intravenous therapy before oral step-down, sample sizes were larger (281 for FQs, 120 for TMP-SMX, and 238 for HBBLs), and HBBLs had a significantly higher risk of recurrence relative to FQs (aHR, 2.23 [95% CI, 1.03–4.83]; *P* = .04) (Supplementary Figure 7). Finally, our 2-group model did not show a significant difference in 60-day recurrence between LBBLs versus HBBLs, although recurrence appeared to be less common among patients receiving LBBLs (Supplementary Figure 8).

### Post Hoc Analysis

Given the unexpected observation of higher 60-day recurrence with short-course FQs (5 of 37 [13.5%] vs 7 of 211 [3.3%] for longer-course FQs; *P* = .02), we conducted a post hoc analysis to explore plausible explanations. We manually re-reviewed patient records of patients receiving FQs to identify risk factors for recurrence. Pairwise comparisons found a significantly higher incidence of previous positive urine cultures (>2 within the past year) among patients in the FQ group with recurrence compared with those without recurrence (3 of 12 [25%] vs 3 of 236 [1%], respectively; *P* = .002) (Supplementary Table 2). Other potentially contributing factors included comorbid conditions (eg, diabetes, heart failure, and liver disease), concomitant prescribing of agents containing multivalent cations, history of kidney stones, and suboptimal FQ dosing, but these differences between the recurrence and nonrecurrence FQ groups were not statistically significant. Finally, we fit a LASSO (Least Absolute Shrinkage and Selection Operator) penalized regression model to further explore these predictors of recurrence. Although the model was limited by low numbers, history of kidney stones and history of multiple recurrent UTIs in the past year were both associated with higher recurrence rates.

Given concerns with low HBBL dosing in our study, we conducted a limited post hoc subgroup analysis of dosing stratified by renal function (using unadjusted data). This suggested that preserved renal function may contribute to HBBL recurrence (Supplementary Table 3) but did not reveal a significant relationship between dosing and recurrence (Supplementary Table 4).

## DISCUSSION

The real-world evidence generated from this large, observational, multicenter cohort study using causal inference methods in a target trial emulation framework provides several important insights on oral step-down therapy for GN-BSI from a urinary tract source. First, we did not observe any significant difference in effectiveness between FQs and TMP-SMX in preventing 60-day recurrence. This is notable, given that most TMP-SMX–treated patients received lower-than-recommended doses (ie, 1 double-strength tablet every 12 hours instead of 2) [20] Our modeling suggested that when TMP-SMX is used as step-down therapy, a total antibiotic duration >8 days may be associated with lower recurrence rates than shorter courses (≤8 days), although the difference was not statistically significant.

We also observed a lower risk of recurrence when FQ step-down regimens were prescribed in longer durations (>8 days). This finding should be interpreted with caution given the efficacy of short-course FQs demonstrated in multiple randomized trials [5, 6, 26, 30], but it does raise a question about real-world factors that may influence FQ effectiveness. In post hoc analyses, we identified several potential contributors to higher recurrence rates that were more prevalent among patients receiving short-course FQ, including history of recurrent UTIs, history of kidney stones, suboptimal dosing, interactions with multivalent cations, and comorbid conditions. Arguably, patients with nonmodifiable risk factors (eg, history of recurrent UTI or kidney stones and other comorbid conditions) could be excluded from the definition of “uncomplicated” GN-BSI, and it is conceivable that longer durations could mitigate recurrence for such patients. However, short-course FQ therapy is reasonable for patients without these factors (ie, those with truly uncomplicated GN-BSI), particularly if dosing is optimized and drug interactions avoided. It was notable that FQs and TMP-SMX had similar rates of adverse events as HBBLs/LBBLs in our data, although detection of these was limited by retrospective data collection. Overall, and in the context of other studies [30, 31], our findings suggest that FQs and TMP-SMX may be preferred options for susceptible isolates, but further study is needed to define optimal dosing and duration.

Conversely, we found that HBBL step-down was associated with >2-fold higher recurrence than FQs, regardless of duration. Although this observation did not reach statistical significance in the primary analysis, it was significant in the larger sensitivity analysis allowing up to 7 days of intravenous therapy before the oral switch. These findings are similar to those of Punjabi et al [16], where BLs had 2-fold higher odds of recurrence than FQ/TMP-SMX (with a median of 3–5 days of intravenous therapy before oral step-down). It is worth noting that the recurrence rate among HBBLs in our cohort was low overall (8%) even though the majority of patients receiving HBBLS received lower-than-recommended dosing. While further study is needed to define optimal HBBL dosing/duration and optimal days of intravenous therapy before oral HBBL step-down, high-dose HBBLs might still be reasonable in some patients (eg, to avoid central catheter placement and outpatient intravenous antibiotics). However, our data suggest that FQs or TMP-SMX should be preferred as oral step-down therapy for GN-BSI.

The advantages of our study (large sample size in an integrated health system, granular data from manual record review, causal inference methods, and capturing recurrent UTI in addition to bacteremia) likely allowed us to detect differences between HBBLs and FQs when other studies did not [9, 10, 13–15]. Future studies should consider these factors because more data are needed to determine how to optimize GN-BSI treatment to reduce recurrences. Our data also identified opportunities for stewardship intervention for uncomplicated GN-BSI, including standardization of oral antibiotic selection across care venues, and shortened treatment durations for patients meeting criteria (our stewardship team is implementing an uncomplicated GN-BSI guideline to address both items).

Our analysis is limited by potential unmeasured confounders that may have influenced prescribing choices (ie, indication bias) and incomplete data that may have contributed to recurrence risk (eg, patient compliance, comorbid conditions, or postdischarge change in antibiotics). We were unable to distinguish between recurrence versus new infection during retrospective review, and documentation of negative/clearance cultures was not required for enrollment in this real-world study; however, sensitivity analyses at days +30, +60, and +90 yielded similar results. Although missingness was rare in our data set, readmissions and recurrences outside our health system were not captured and may have led to underreporting of outcomes. In our observational data, prescribing patterns determined the degree of variation available for study.

We were unable to control for provider-level or facility-level variability owing to sample size, and most patients received a total treatment duration of 10–14 days; thus, we were unable to draw strong conclusions regarding shorter durations by antibiotic class. Our analysis of uncomplicated GN-BSI limited the study patients to only 25% (648 of 2571) of the screened population, which limits external validity, and highlights the need for more data in complicated GN-BSI. Most notably, our ability to evaluate HBBL effectiveness was limited by current susceptibility testing practices, including lack of granular MICs, use of surrogate intravenous antibiotics to infer susceptibility of oral agents, and lack of systemic susceptibility breakpoints for oral BLs. Our unexpected findings of lower recurrence rates with LBBLs compared with HBBLs might also have been affected by these issues, which should all be considered in future studies.

In conclusion, our data suggest that FQs and TMP-SMX are preferred oral step-down choices for uncomplicated GN-BSI from a urinary tract source when feasible (ie, depending on toxicity risk and isolate susceptibility). Shorter courses of therapy may be suboptimal for patients with key risk factors for recurrence, although additional studies are needed. HBBLs were associated with higher recurrence rates regardless of treatment duration but were affected by suboptimal dosing and limitations of current susceptibility testing practices. Further studies are needed to validate systemic susceptibility breakpoints for oral BLs and to determine whether aggressive dosing and/or extended treatment duration can mitigate the higher risk of recurrence observed with HBBLs.

## Supplementary Material

ofad657_Supplementary_Data

## Supplementary Data

Supplementary materials are available at *Open Forum Infectious Diseases* online. Consisting of data provided by the authors to benefit the reader, the posted materials are not copyedited and are the sole responsibility of the authors, so questions or comments should be addressed to the corresponding author.
