# Correction to: Oral β-Lactams, Fluoroquinolones, or Trimethoprim-Sulfamethoxazole for Definitive Treatment of Uncomplicated *Escherichia coli* or *Klebsiella* Species Bacteremia From a Urinary Tract Source

**DOI:** 10.1093/ofid/ofae191

**Published:** 2024-04-09

This is a correction to: Sameer Alzaidi, John J Veillette, Stephanie S May, Jared Olson, Katarina Jackson, C Dustin Waters, Allison M Butler, Mary A Hutton, Whitney R Buckel, Brandon J Webb, Oral β-Lactams, Fluoroquinolones, or Trimethoprim-Sulfamethoxazole for Definitive Treatment of Uncomplicated *Escherichia coli* or *Klebsiella* Species Bacteremia From a Urinary Tract Source, *Open Forum Infectious Diseases*, Volume 11, Issue 2, February 2024, ofad657, https://doi.org/10.1093/ofid/ofad657.

The following clarificatory sentence has been added to the legends of figures 2 and 3: ‘Data represent cumulative incidence curves generated from the propensity-weighted Cox proportional hazards models.’

