# Supplementary material for: Oral β-Lactams, Fluoroquinolones, or Trimethoprim-Sulfamethoxazole for Definitive Treatment of Uncomplicated Escherichia coli or Klebsiella Species Bacteremia From a Urinary Tract Source
Source: Open Forum Infect Dis. 2023 Dec 27;11(2):ofad657. doi: 10.1093/ofid/ofad657 (PMC10873539; doi:10.1093/ofid/ofad657)
Supplement: ofad657_Supplementary_Data [file ofad657_supplementary_data.zip › Uncomp GNB revised Supplemental Material 11-20-23.docx]

**Supplemental Material**

**Table S1. Balance Table for the Propensity Score Model**

| Standardized effect size | Minimum p-value | Maximum Kolmogorov-Smirnov test statistic | Minimum Kolmogorov-Smirnov p-value | Stop Method |
| --- | --- | --- | --- | --- |
| 0.7056 | 0 | 0.227 | 0.0014 | Unweighted |
| 0.4574 | 0 | 0.1733 | 0.1016 | ES Mean* |
| 0.4574 | 0 | 0.1733 | 0.1016 | KS Mean |

* Both stop methods performed similarly. ES Mean weights was selected based on the absolute standardized mean differences, minimum p-values, and effective sample sizes.

**Figure S1. Propensity Score Box Plots to Assess Overlap**


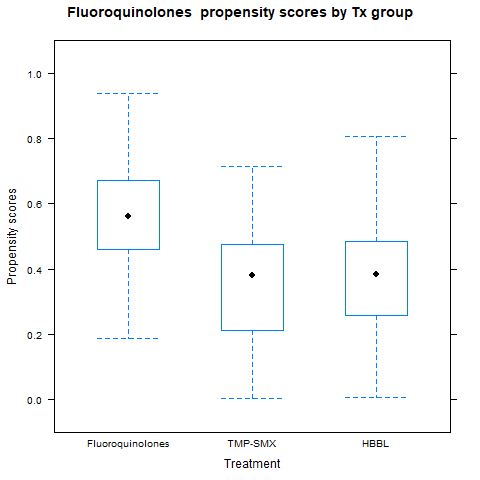

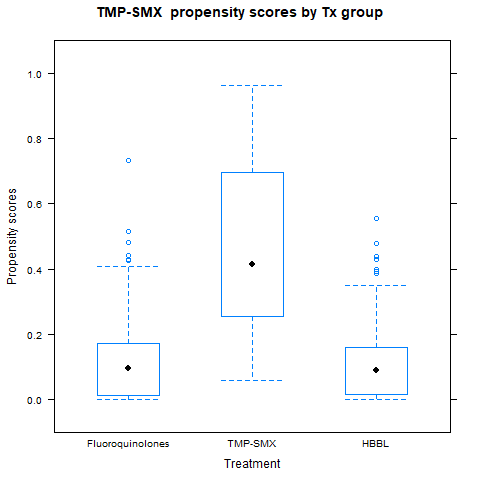

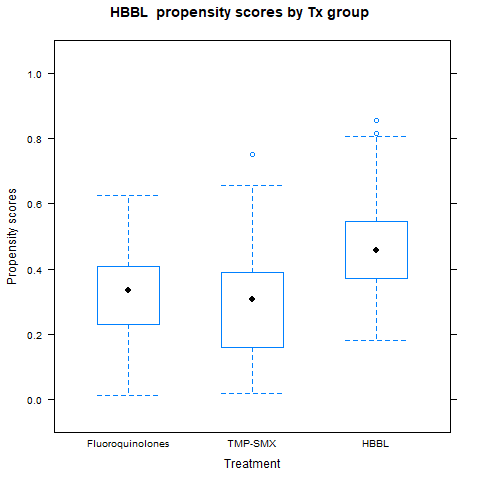


**Figure S2. Youden Index and Associated ROC Curve**


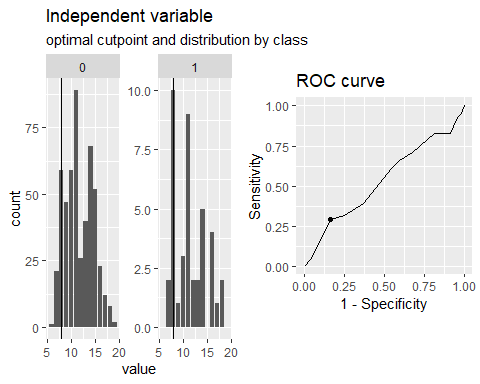

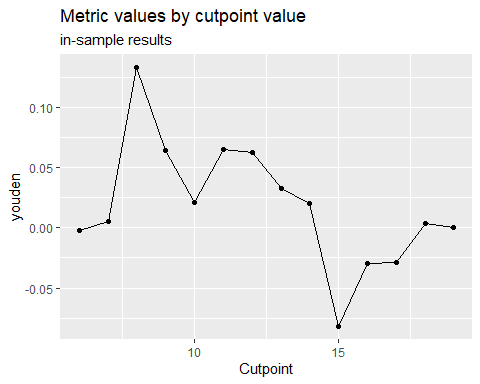


8-day Treatment Duration

**Youden Index**

6 7 8 9 10 11 12 13 14 15 16 17 18 19

**Total Treatment Duration (Days)**

**Figure S3. Recurrence-free days (through day 30) for GN-BSI based on oral stepdown antibiotic**


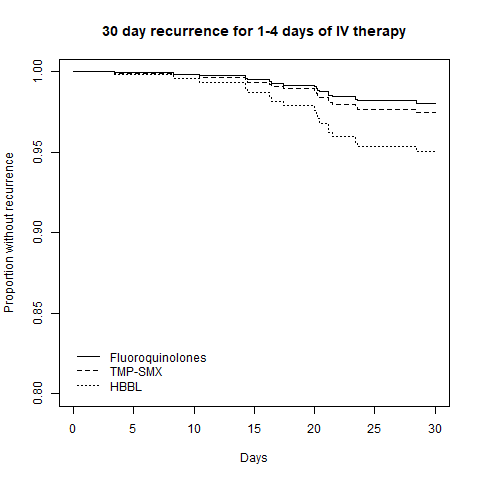


| **Number of**  **Patients** | **Variable** | **Adjusted Hazard Ratio**  **(95% CI)** | **P-value** |
| --- | --- | --- | --- |
| 248 | Fluoroquinolones (referent) | - | - |
| 99 | Trimethoprim-sulfamethoxazole (TMP-SMX) | 1.30 (0.28, 5.95) | 0.739 |
| 201 | High bioavailability β-lactams (HBBL) | 2.60 (0.82, 8.19) | 0.103 |
| -- | Days of IV antibiotics | 1.05 (0.66, 1.68) | 0.834 |
| -- | Total days of IV and oral antibiotics | 1.07 (0.90, 1.27) | 0.455 |

**Figure S4. Recurrence-free days (through day 90) for GN-BSI based on oral stepdown antibiotic**


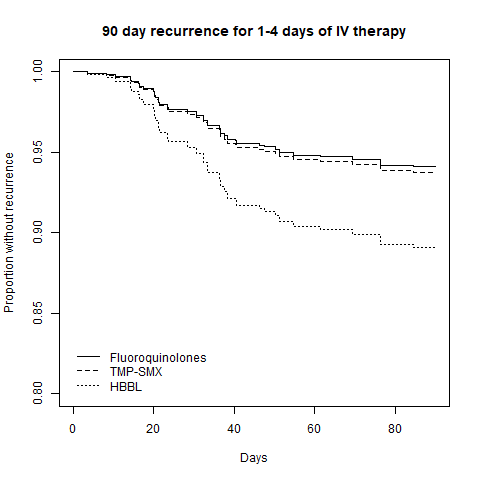


| **Number of**  **Patients** | **Variable** | **Adjusted Hazard Ratio**  **(95% CI)** | **P-value** |
| --- | --- | --- | --- |
| 248 | Fluoroquinolones (referent) | - | - |
| 99 | Trimethoprim-sulfamethoxazole (TMP-SMX) | 1.06 (0.37, 3.04) | 0.917 |
| 201 | High bioavailability β-lactams (HBBL) | 1.90 (0.88, 4.12) | 0.103 |
| -- | Days of IV antibiotics | 1.00 (0.69, 1.44) | 0.996 |
| -- | Total days of IV and oral antibiotics | 1.01 (0.90, 1.14) | 0.851 |

**Figure S5. Recurrence-free days (through day 60) for GN-BSI based on oral stepdown antibiotic – ALL Beta-lactams**


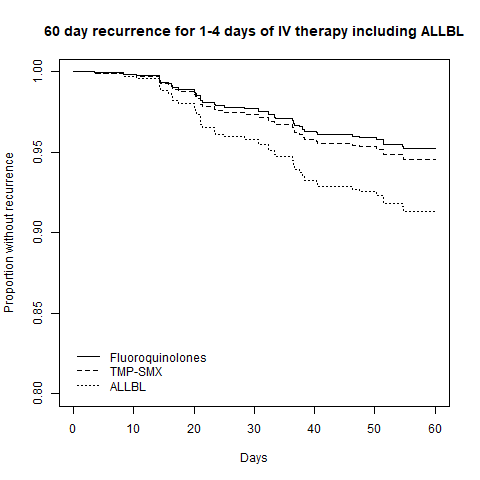


| **Number of**  **Patients** | **Variable** | **Adjusted Hazard Ratio**  **(95% CI)** | **P-value** |
| --- | --- | --- | --- |
| 248 | Fluoroquinolones (referent) | - | - |
| 99 | Trimethoprim-sulfamethoxazole (TMP-SMX) | 1.15 (0.36, 3.62) | 0.817 |
| 301 | All β-lactams (ALLBL):  HBBL (n = 201) + LBBL (n = 100) | 1.85 (0.85, 4.03) | 0.119 |
| -- | Days of IV antibiotics | 0.97 (0.73, 1.29) | 0.854 |
| -- | Total days of IV and oral antibiotics | 0.97 (0.85, 1.10) | 0.641 |

**Figure S6. Recurrence-free days (through day 60) for GN-BSI based on oral stepdown antibiotic (low cefazolin MIC HBBLs)**

| **Number of**  **Patients** | **Variable** | **Adjusted Hazard Ratio**  **(95% CI)** | **P-value** |
| --- | --- | --- | --- |
| 248 | Fluoroquinolones (referent) | - | - |
| 99 | Trimethoprim-sulfamethoxazole (TMP-SMX) | 0.91 (0.30, 2.76) | 0.864 |
| 165 | HBBL and blood isolate was susceptible  at cefazolin MIC ≤ 2 mg/L | 1.93 (0.81, 4.55) | 0.135 |
| -- | Days of IV antibiotics | 1.07 (0.74, 1.54) | 0.727 |
| -- | Total days of IV and oral antibiotics | 0.96 (0.84, 1.11) | 0.608 |


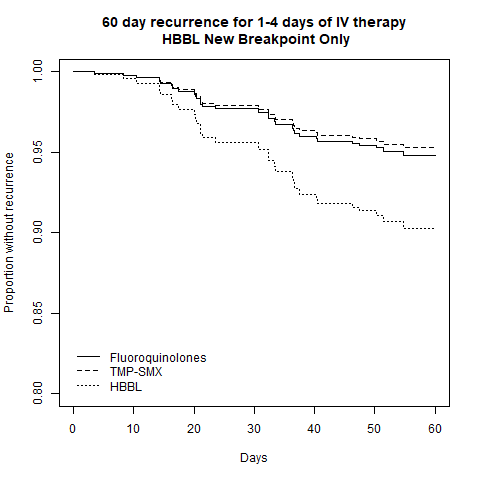


**Figure S7. Recurrence-free days (through day 60) for GN-BSI based on oral stepdown antibiotic**

**(allowing 1-7 days IV therapy prior to oral stepdown)**


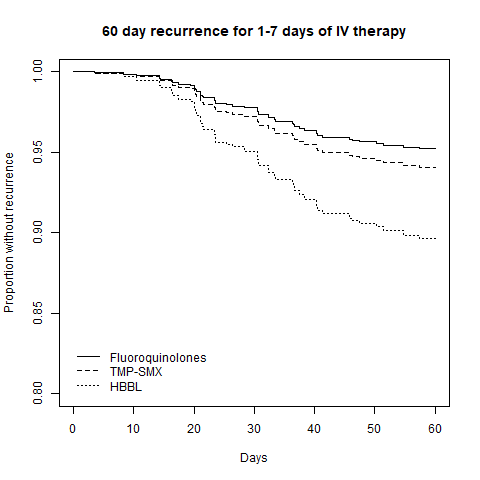


| **Number of**  **Patients** | **Variable** | **Adjusted Hazard Ratio**  **(95% CI)** | **P-value** |
| --- | --- | --- | --- |
| 281 | Fluoroquinolones (referent) | - | - |
| 120 | Trimethoprim-sulfamethoxazole (TMP-SMX) | 1.24 (0.47, 3.32) | 0.662 |
| 238 | High bioavailability β-lactams (HBBL) | **2.23 (1.03, 4.83)** | **0.043** |
| -- | Days of IV antibiotics | 1.14 (0.91, 1.44) | 0.249 |
| -- | Total days of IV and oral antibiotics | 0.99 (0.87, 1.11) | 0.809 |

**Figure S8. Recurrence-free days (through day 60) for GN-BSI based on oral stepdown antibiotic (HBBL vs LBBLs)**


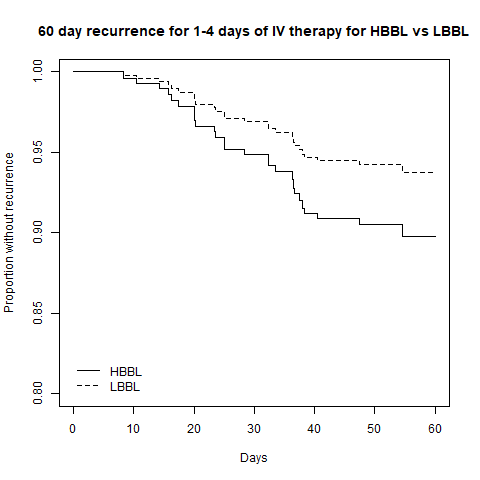


| **Number of**  **Patients** | **Variable** | **Adjusted Hazard Ratio**  **(95% CI)** | **P-value** |
| --- | --- | --- | --- |
| 201 | High bioavailability β-lactams (HBBL)  (referent) | - | - |
| 100 | Low bioavailability β-lactams (LBBL) | 0.59 (0.23, 1.55) | 0.289 |
| -- | Days of IV antibiotics | 0.89 (0.62, 1.28) | 0.527 |
| -- | Total days of IV and oral antibiotics | 1.02 (0.88, 1.17) | 0.834 |

**Table S2. Unadjusted Descriptive Statistics for Fluoroquinolone Patients Stratified by Recurrence**

| **Variable** | **No recurrence**  **(n = 236)** | **60-day recurrence**  **(n = 12)** | **P-value** |
| --- | --- | --- | --- |
| Admit year^1^ |  |  |  |
| 2016 | 11 (5) | 0 (0) |  |
| 2017 | 24 (10) | 1 (8) |  |
| 2018 | 27 (11) | 1 (8) |  |
| 2019 | 55 (23) | 3 (25) |  |
| 2020 | 57 (24) | 4 (33) |  |
| 2021 | 36 (15) | 0 (0) |  |
| 2022 | 26 (11) | 3 (25) |  |
| Age (years), median (IQR) | 64.0 (45.0-76.0) | 65.5 (33.5-74.0) |  |
| Female | 199 (84) | 10 (83) |  |
| Weight (kg), median (IQR) | 81.2 (68.2-98.6) | 78.1 (70.8-100.5) |  |
| Diabetes | 95 (40) | 7 (58) | 0.240 |
| Heart failure | 40 (17) | 3 (25) | 0.442 |
| Liver disease | 46 (19) | 5 (42) | 0.075 |
| Chronic kidney disease, stage II or higher | 51 (22) | 2 (17) |  |
| History of kidney stones | 8 (3) | 2 (17) | 0.098 |
| >2 Positive urine cultures in past year  (surrogate for previous UTIs) | 3 (1) | 3 (25) | **0.002** |
| Charlson Score, median (IQR) | 4.0 (2.0-8.0) | 5.0 (1.8-7.0) |  |
| Pitt bacteremia score, median (IQR) | 1.0 (1.0-3.0) | 1.5 (1.0-3.0) |  |
| Co-administration of multivalent cation | 76 (32) | 6 (50) | 0.219 |
| Adequate therapy within 12h | 232 (98) | 11 (92) |  |
| Index blood culture organism |  |  |  |
| Escherichia coli | 221 (94) | 10 (83) |  |
| Klebsiella | 15 (6) | 2 (17) |  |
| Admitted to intensive care unit | 26 (11) | 2 (17) |  |
| Received vasopressors | 10 (4) | 0 (0) |  |
| CrCl<30 | 15 (6) | 0 (0) |  |
| CrCl<60 | 102 (43) | 6 (50) |  |
| CrCl<120 | 215 (91) | 11 (92) |  |
| Time to clinical stability from index blood culture (days), median (IQR) | 0.6 (0.03-1.3) | 0.5 (0.1-1.6) |  |
| Received recommended dosing | 139 (59) | 4 (33) | 0.131 |
| Days of IV antibiotics, median (IQR) | 3.0 (2.0-4.0) | 2.5 (2.0-3.0) | 0.201 |
| Days of oral antibiotics, median (IQR) | 10.0 (7.0-11.0) | 7.0 (5.8-7.8) | **0.034** |
| Days of IV and oral antibiotics, median (IQR) | 12.0 (10.0-14.0) | 9.5 (8.0-10.2) | **0.014** |
| Ciprofloxacin new breakpoint susceptible | 227 (96) | 12 (100) |  |
| Levofloxacin new breakpoint susceptible | 236 (100) | 12 (100) |  |

^1^Values are presented as number (percent), unless otherwise stated

**Table S3. HBBL Stepdown Regimens and Unadjusted 60-day Recurrence Stratified by Renal Function**

| **Creatinine Clearance (mL/min) at Discharge** | **< 30**  **(n = 18)** | **30-50**  **(n = 51)** | **> 50**  **(n = 132)** |
| --- | --- | --- | --- |
| **Outcomes** |  |  |  |
| Recurrence at 60 days | 1 (6) | 4 (8) | 13 (10) |
| **Oral antibiotic dosing** |  |  |  |
| Amoxicillin |  |  |  |
| 500 – 1,000 mg q8h | -- | 5 (10) | 11 (8) |
| 500 – 1,000 mg q12h | 3 (17) | 4 (8) | 5 (4) |
| Amoxicillin-clavulanic acid |  |  |  |
| 875 mg q12h | 2 (11) | 7 (14) | 26 (20) |
| Cephalexin |  |  |  |
| 500 – 1,000 mg q6h | 2 (11) | 10 (20) | 68 (52) |
| 500 – 1,000 mg q8h | 8 (44) | 22 (43) | 17 (13) |
| 500 – 1,000 mg q12h | 3 (17) | 3 (6) | 5 (4) |

**Table S4. Oral Stepdown Regimens Stratified by Dosing and Unadjusted 60-day Recurrence**

| **HBBL patients (n = 201)** | **No Recurrence** | **Recurrence** | **P-value^1^** |
| --- | --- | --- | --- |
| **Recommended HBBL Dosing**  **(n = 60)** | 53 (88%) | 7 (12%) | 0.25 |
| **Lower HBBL Dosing**  **(n = 141)** | 132 (94%) | 9 (6%) |  |
|  |  |  |  |
| **FQ patients (n = 248)** | **No Recurrence** | **Recurrence** | **P-value^1^** |
| **Recommended FQ Dosing**  **(n = 146)** | 142 (97%) | 4 (3%) | 0.08 |
| **Lower FQ Dosing**  **(n = 102)** | 94 (92%) | 8 (8%) |  |
|  |  |  |  |
| **TMP-SMX patients (n = 99)** | **No Recurrence** | **Recurrence** | **P-value^1^** |
| **Recommended TMP-SMX Dosing**  **(n = 3)** | 3 (100%) | 0 (0%) | 1.00 |
| **Lower TMP-SMX Dosing**  **(n = 96)** | 88 (92%) | 8 (8%) |  |

^1^By Fisher’s exact test
