# Supplementary material for: Oral β-Lactams, Fluoroquinolones, or Trimethoprim-Sulfamethoxazole for Definitive Treatment of Uncomplicated Escherichia coli or Klebsiella Species Bacteremia From a Urinary Tract Source
Source: Open Forum Infect Dis. 2023 Dec 27;11(2):ofad657. doi: 10.1093/ofid/ofad657 (PMC10873539; doi:10.1093/ofid/ofad657)
Supplement: ofad657_Supplementary_Data [file ofad657_supplementary_data.zip › GNB Data Collection Tool (REDCap) - Appendix.pdf]

# Demographics

Record ID ______________________

Patient EMPI (MRN) ______________________

Account number (FIN number) ______________________

**SCREEN for potential exclusion:
Was blood culture collected > 48 hours after admission? ______________________

NOTE: If this number is > 48 hours, patient should be excluded (finish this page, change record to 'Complete' and proceed to next patient).

**SCREEN for potential exclusion:
Did patient die prior to discharge? ______________________

EXCLUDE patient if number is NEGATIVE or ZERO. If positive or blank, continue data collection

**SCREEN for potential exclusion:
Total organisms in the blood culture ______________________

NOTE: If this number is 2 or more, patient should be excluded (finish this page, change record to 'Complete', and proceed to next patient).

**SCREEN for potential exclusion:
Total organisms in the urine ______________________

NOTE: If this number is 2 or more, patient should be excluded (finish this page, change record to 'Complete', and proceed to next patient).

**SCREEN for potential exclusion:
Length of stay in days ______________________

Note - complete this page and EXCLUDE if LOS > 14 days (e.g. 15 or more)

Discharge disposition ______________________

Please select the highest level of care

**IF the selected FIN is an Emergency Department ("ED") encounter**
-Review list of subsequent encounters in iCentra to see if pt was brought back for subsequent ED visits or was admitted to hospital.
-If pt was admitted, change highest level of care to "inpatient", and use info from that admission to revise the admit/discharge dates below. Double check that the abx info on the next few pages is correct based on the inpatient admission

__________________________________

**SCREEN for potential exclusion:
Was patient discharged to either of the following?
-Hospice
-A non-Intermountain INPATIENT facility (Discharge disposition field will say "Acute Facility - Non-IHC"). NOTE: Discharge to Long Term Acute Care (LTAC) or Skilled Nursing facility (SNF) is OK to include!

If discharged to hospice or transferred to non-Intermountain INPATIENT facility, mark YES, complete this page, and EXCLUDE patient from further analysis.

○ Yes
○ No

Was patient lost to follow-up?
(i.e. NO known encounters or patient contact in iCentra after discharge from hospital or ED)

○ Yes
○ No

**SCREEN for potential exclusion:
Was the bacteremia due to a source OTHER than UTI?

NOTES:
Read the Discharge summary note - did they say the bacteremia was from UTI/pyelo, sepsis due to urinary source, or urosepsis? Or did they mention another source? If another source, EXCLUDE the patient! If UTI, continue with data collection

○ Yes
○ No

**SCREEN for potential exclusion:
Did the patient have an infection in ADDITION TO UTI/bacteremia that influenced antibiotic prescribing?

Read the Discharge Summary note and look through the discharge diagnoses. Did patient receive antibiotics for anything besides UTI? Or did any complicating factors cause the team to prescribe longer than a 2-week course?

-Examples include: prostatitis, epididymitis/orchitis, cellulitis, pneumonia, abscess, septic arthritis, osteomyelitis, or other infections

○ Yes
○ No

| | |
|---|---|
| **SCREEN for potential exclusion:<br>Pregnant? If yes, complete this page and EXCLUDE from further analysis<br><br>NOTES:<br>-STEP 1. Review fields below for Age and Sex. If patient is MALE or is a FEMALE > 50 years old, mark NO and continue data collection<br>-STEP 2. If patient is FEMALE < 50 years old, check lab results for pregnancy screening during the 9 months PRIOR to admission<br>-STEP 3. If no pregnancy testing was performed, review the H&P note (or progress notes if no H&P listed) to confirm patient is not pregnant<br>-->Review the note for any mention of pregnancy ("Patient is a 27-year-old G2 P1 001 at 24 weeks 2 days") | ◯ Yes<br>◯ No |
| Immunocompromised?<br><br>-Review the H&P for immunocompromising conditions:<br>-SOT/BMT<br>-Cancer receiving chemotherapy in last 30 days<br>-HIV/AIDS (If listed, check Lab Extended for last CD4 count to see if < 200.)<br>-Neutropenia (as mentioned in H&P) or absolute neutrophil count < 500 (check CBC w/ diff in H&P or Labs Extended)<br><br>Check H&P for immunocompromising medications:<br>-Anti-rejection (cyslosporine, tacrolimus, sirolimus, mycophenolate mofetil, or mycophenolate sodium)<br>-TNF inhibitors (infliximab, adalimumab, etanercept, certolizumab, golimumab)<br>-DMARDs (Methotrexate, sulfasalazine, mesalamine, hydroxychloroquine, leflunomide, azathioprine)<br>-Steroids (prednisone ≥ 20mg, hydrocortisone ≥ 80mg, methylprednisolone ≥ 16mg, dexamethasone ≥ 3mg) | ☐ Solid organ transplant<br>☐ Bone marrow transplant<br>☐ Chemotherapy within last 30 days<br>☐ Immunosuppressive medications (anti-rejection, TNF inhibitors, DMARDS) within last 30 days<br>☐ Chronic steroids (prednisone equivalents of >=20 mg/day) for >=7 days as a home medication or inpatient medication<br>☐ HIV and CD4 count < 200 cells/microL<br>☐ ANC < 500 cells/microL during index admission |
| Blood culture facility ID | ______________________________ |
| Sex<br><br>NOTE:<br>-if pre-populated, no action needed!<br>-if blank, please fill in | ______________________________ |
| Race | ______________________________ |
| Ethnicity | ______________________________ |
| Age in Years | ______________________________ |
| Age collected text (this will be important for children less than 12 months of age) | ______________________________ |

Height in cm on admission

NOTE:
-if pre-populated, no action needed!

***let's get this via MD Clone for the blanks***
Should be straightforward to pull

______________________

Weight in kg on admission

______________________

Ideal Body Weight

______________________

BMI

______________________

Allergies to Antibiotics
(pre-populated! No action needed on this section)

- ☐ No Known Allergies
- ☐ Penicillin (amoxicillin, ampicillin, oxacillin, nafcillin, piperacillin-tazobactam)
- ☐ Cephalosporin (cephalexin, cefuroxime, cefdinir, ceftriaxone ceftazidime, cefepime)
- ☐ Fluoroquinolone (ciprofloxacin, levofloxacin, moxifloxacin)
- ☐ Trimethoprim/sulfa (or brand names Bactrim, Septra)
- ☐ Nitrofurantoin (or brand name Macrobid)
- ☐ Carbapenem (ertapenem, meropenem, imipenem)

***Penicillin, cephalosporin, FQ, or TMP-SMX allergy***

______________________

Admission date

______________________

Discharge date

______________________

Length of stay in hours

______________________

financial group (if blank, just leave blank)

______________________

Reason for visit

______________________

Admitted from the ER

- ◯ Yes
- ◯ No

Immunocompromised indicator

______________________

# H&P + Discharge notes

## Please review the H&P and the discharge note for the following information

Charlson Comorbidity Score

__________________________________

Specific Comorbidities. Please select for the following:

Review History & Physical note and Discharge note
-consider "Control + F" search function for key terms

NOTE: Some comorbidities (e.g. urinary retention, fistula, cystocele) are discovered on imaging during the acute hospitalization and are assumed to be present at the time of admission. When reviewing the imaging results below, please return here and fill out the checkboxes as needed

- ☐ Diabetes
- ☐ Heart failure with reduced or preserved ejection fraction
- ☐ Chronic liver disease
- ☐ Chronic kidney disease
- ☐ Enlarged prostate/benign prostatic hyperplasia
- ☐ Neurogenic bladder
- ☐ Urinary incontinence
- ☐ Urinary retention
- ☐ Cystocele
- ☐ Bladder outlet obstruction
- ☐ Nephrolithiasis (kidney stone, renal calculi, ureteral stone, ureterolithiasis)
- ☐ Other urologic abnormalities (e.g. fistula, neobladder, urethral sling, urethral stricture, urethral cancer, bladder cancer, hydrocele, etc)

Other urologic abnormality. Please describe

__________________________________

Please select any that were present AT BASELINE when patient was admitted for bacteremia. Manual review.

(e.g. patient had indwelling suprapubic cath when admitted to hospital for bacteremia)

-Review H&P
-consider "Control + F" search function for key terms

- ☐ Foley catheter
- ☐ Suprapubic catheter
- ☐ Ureteral stent
- ☐ Nephrostomy tube
- ☐ Intermittent self-catheterization ("self-cath" or "straight cath")
- ☐ Recent urologic procedure in last 2 weeks (e.g. prostate biopsy, TURP, cystoscopy, bladder irrigation, urinary stent or tube exchange)

## Imaging during hospitalization

CT or MRI imaging of abdomen/pelvis (including CT Stone Protocol)?

- ○ Yes
- ○ No

Ultra-sound imaging (abdominal or retroperitoneal)?

- ○ Yes
- ○ No

Please select any of the following if true during the hospitalization.

Requires manual review of the CT, MRI, or ultrasound imaging for: Hydronephrosis, abscess, or renal/ureteral stones
-Go to Results Review --> Imaging. Right click in the grey bar to Change search Criteria to start/end dates for the hospitalization. Double click on imaging results, scroll down to read the IMPRESSION.

-Also review the Discharge summary note for any of these items!

- ☐ Hydronephrosis (prepolulated with ICD codes, but needs a manual double check!)
- ☐ Underwent procedure for ureteral stent or nephrostomy tube (**check Discharge Summary under "Procedures" for mention of stent or nephrostomy tube placement. Can also do Control + F for "stent" or "tube")
- ☐ Perinephric abscess/Renal abscess (prepopulated with ICD codes)
- ☐ Renal calculi (Prepopulated with ICD codes) - also called ureteral stones, ureterolithiasis, kidney stones, renal stones, nephrolithiasis

## Severity of illness

Pitt Bacteremia score (worst reading on the day of index BCx):

Manual review:
TEMPERATURE and BLOOD PRESSURE
-Go to Results Review, Vitals-Recent and change date range to 1 day BEFORE admission and 1 day AFTER discharge dates. In Table view, graph Systolic blood pressure (SBP) and Temperature.

MENTAL STATUS and RESPIRATORY
-Change Flowsheet to Nursing View (Group view) - Look at Neuro Check under Orientation for mental status (need to scroll down to values recorded over the first 24 hours of admission) Was the patient disoriented? Comatose? We also recommend checking the H&P for a description of the patient's mental status (especially if nursing documentation is unclear).
-In the same view, look at RESPIRATORY to see if patient required mechanical ventilation/intubation (may need to scroll down)

CARDIAC STATUS
-Review Discharge Summary to see if it mentions the patient having a Cardiac arrest (check diagnoses and text)

**SCORING**
TEMPERATURE
---- ≤35 or ≥40°C: 2 points
---- 35.1-36°C or 39.0- 39.9°C: 1 point
BLOOD PRESSURE (hypotension: 2 points)
----SBP < 90 mmHg
MENTAL STATUS [disorientation (GCS 9-14): 1 point, stupor (GCS 4-8): 2 points, coma (GCS 3): 4 points]
RESP STATUS (mechanical ventilation: 2 points)
CARDIAC STATUS (cardiac arrest within 3 days of positive BCx: 4 points)

________________________________

ICU admission? ***pull via MDClone***

- ◯ Yes
- ◯ No

| | |
|---|---|
| Total ICU stay (round to full days) ***pull from MDClone!***<br><br>In the Encounter Location History Viewer, subtract date of transfer to floor from date of admission to ICU | ________________________ |
| Received vasopressors within 24 hours of bacteremia? ***pull from MDClone***<br><br>-Check for vasopressors in MAR summary. Change Start/End dates, filter by Therapeutic Class View, and click on cardiovascular agents. Look for norepinephrine, epinephrine, or vasopressin. | ◯ Yes<br>◯ No |
| Maximum temperature in celsius | ________________________ |
| Lowest temperature in celsius | ________________________ |
| Minimum diastolic blood pressure | ________________________ |
| minimum systolic blood pressure | ________________________ |
| highest heart rate | ________________________ |
| Highest respiratory rate | ________________________ |
| Highest WBC count | ________________________ |
| Lowest WBC count | ________________________ |
| Highest lactate | ________________________ |
| Highest serum creatinine within 24 hours of ADMISSION | ________________________ |
| Last serum Creatinine within 24 hours of DISCHARGE<br><br>NOTE:<br>-if this field is pre-populated, no action necessary!<br>-if this field is BLANK, please fill in<br><br>***might need an MDClone data pull to do this if we really need it*** | ________________________ |

Lowest estimated creatinine clearance (CrCl) within 24 hrs of ADMISSION

Pre-calculated value

______________________________

Lowest estimated CrCl within 24 hours of DISCHARGE.

NOTE:
-If this field is pre-populated, no action necessary!
-If this field is blank, just leave blank. You only need to fill in the field above for "Highest serum creatinine within 24 hours of DISCHARGE". Then continue with data collection! We will perform the calculations later.

______________________________

Urine WBC

______________________________

Leukocyte esterase in urine

______________________________

nitrites

______________________________

bacteria in urine

______________________________

Complicated indicator

______________________________

# Microbiology

| | |
|---|---|
| Index blood culture date time stamp | ________________________ |
| Index blood culture organism | ________________________ |
| Total blood cultures collected 12 hours before and 12 hours after the first positive blood culture | ________________________ |
| Order location of the blood culture | ________________________ |
| First organism in urine | ________________________ |
| Urine cx dts | ________________________ |
| CFU in Urine Cultures | ________________________ |
| History of Recurrent UTI (leave blank, pre-populated!):<br>Number of prior positive urine cultures within the last 365 days up to 2 days prior to the first positive blood culture.<br><br>Positive urine culture is defined as growth of at least 10^5 CFU of at least one uropathogen: Escherichia coli, Pseudomonas aeruginosa, Enterococcus, Klebsiella, Enterobacter, Proteus, Citrobacter, Providencia, Morganella, or Serratia spp. | ________________________ |
| Repeat Blood cultures collected 24-48 hours after first blood culture?<br>(Pre-populated - leave blank if blank!) | ◯ no blood culture obtained<br>◯ Blood culture obtained and no growth<br>◯ Blood collected and positive for the organism<br>◯ Blood collected and positive for new organism (even if both new and old organism are positive) |
| Repeat Blood cultures collected 48-72 hours after first blood culture?<br>(Pre-populated - leave blank if blank!) | ◯ no blood culture obtained<br>◯ Blood culture obtained and no growth<br>◯ Blood collected and positive for the organism<br>◯ Blood collected and positive for new organism (even if both new and old organism are positive) |
| Repeat Blood cultures collected 72 hours to 7 days after first blood culture?<br>(Pre-populated - leave blank if blank!) | ◯ no blood culture obtained<br>◯ Blood culture obtained and no growth<br>◯ Blood collected and positive for the organism<br>◯ Blood collected and positive for new organism (even if both new and old organism are positive) |

| | |
|---|---|
| Repeat Blood cultures collected 7-14 days after first blood culture?<br>(Pre-populated- leave blank if blank!) | ◯ no blood culture obtained<br>◯ Blood culture obtained and no growth<br>◯ Blood collected and positive for the organism<br>◯ Blood collected and positive for new organism (even if both new and old organism are positive) |
| Repeat Blood cultures collected 14-90 days after first blood culture?<br>(Pre-populated- leave blank if blank!) | ◯ no blood culture obtained<br>◯ Blood culture obtained and no growth<br>◯ Blood collected and positive for the organism<br>◯ Blood collected and positive for new organism (even if both new and old organism are positive) |

*****Review this field for primary outcomes of the study!*****
**If the field says "Urine positive for same organism" there is additional information to fill in. If not (if it contains different text or is blank) move on to next field!**

| | |
|---|---|
| **OUTCOMES**<br>Repeat URINE culture positive for SAME index pathogen between 4 and 90 days? | ◯ no culture<br>◯ culture obtained but not growing pathogen in question<br>◯ urine positive for same organism |
| Date of the first positive urine culture growing the SAME index pathogen (from 4 to 90 days)<br><br>NOTES:<br>1. Go to Results Review --> Microbiology<br>2. Change the start date to the day prior to index admission<br>3. Change the end date to at least 90 days/3 months after discharge<br>4. Record the date and time of the first urine culture with matching organism | ________________________________ |
| Was the positive urine culture associated with a readmission to the ED or hospital?<br><br>Notes:<br>-Click the patient's FIN hyperlink (in upper righthand corner of the chart) to bring up a list of previous encounters. Look for either an Emergency or Inpatient encounter around the time the urine culture was obtained. If you find an ED or Inpatient encounter, mark YES. If not, mark NO | ◯ Yes<br>◯ No |

| | |
|---|---|
| Did patient have urinary symptoms at time of urine culture?<br><br>Review notes in iCentra for documentation of UTI symptoms:<br>-Check the Notes section in iCentra. Set the start date to a few days before and end date to 4-5 days after the urine culture was ordered. Filter by Date instead of by Note type.<br>-Look for any notes where the patient's symptoms may have been documented (this would be the H&P and/or ED note if patient was readmitted, or any outpatient clinic or communication/Phone message notes if the UTI was treated as outpatient)<br><br>Regarding symptoms:<br>- If there is any mention of symptoms such as dysuria, urgency, frequency, suprapubic pain, flank pain, fever, or sepsis from suspected urinary source, mark YES<br>-If patient had non-specific symptoms such as abdominal pain, nausea/vomiting, or altered mental status with no alternative cause or unknown cause, then mark YES | ◯ yes<br>◯ no<br>◯ unknown |
| Clinical recurrence: Was patient treated with antibiotics for a diagnosis UTI?<br><br>NOTE:<br>-Read the H&P, Discharge Summary, and/or ED note corresponding to the urine culture date. If they mention giving antibiotics for UTI in the note (or sepsis from suspected urinary source) then answer YES.<br>-If the notes are unclear regarding antibiotics and/or indication, you can also check MAR summary. Right click in the grey bar to change the start/end dates to a few days before and after the urine culture date. Click on the purple arrow (upper lefthand corner), then change from "Time View" to "Therapeutic Class" view. Select Anti-infectives. Look at the indication on the antibiotic orders. | ◯ Yes<br>◯ No<br>◯ Unknown |
| Time to recurrent UTI | ______________________ |
| ID Pharm double check?? | ◯ Yes<br>◯ No |

*****Review this field for primary outcomes of the study!*****
**If the field says "Blood culture positive for same pathogen" there is additional information to fill in. If not (if it contains different text or is blank) move on to next field!**

| | |
|---|---|
| **OUTCOMES**<br>Repeat BLOOD culture positive for SAME index pathogen between 4 and 90 days? | ◯ no blood culture<br>◯ blood culture obtained but not growing pathogen in question<br>◯ blood culture positive for same pathogen |
| Date of the first positive blood culture growing the SAME index pathogen (from 4 to 90 days) | ______________________ |

Time to recurrent bacteremia (days) ________________________________

***Recurrence??*** ________________________________

***Time to Recurrence*** ________________________________

***Recurrence within 30 days?*** ________________________________

***Recurrence within 60 days?*** ________________________________

***Recurrence within 90 days?*** ________________________________

***The rest of this page contains Pre-populated data that you do NOT need to fill in***

| | |
|---|---|
| Repeat URINE culture positive for DIFFERENT pathogen between 4 and 90 days? | ◯ no culture<br>◯ culture obtained but negative<br>◯ urine positive for DIFFERENT pathogen<br>◯ N/A - same pathogen |
| Repeat BLOOD culture positive for DIFFERENT pathogen between 4 and 90 days? | ◯ no culture<br>◯ culture obtained but negative<br>◯ blood culture positive for DIFFERENT pathogen<br>◯ N/A - same pathogen |
| Ampicillin: Worst susceptibility from urine and blood collected during the first 24 hours of the first blood culture | ◯ resistant<br>◯ susceptible |
| Amp/sulbactam: Worst susceptibility from urine and blood collected during the first 24 hours of the first blood culture | ◯ resistant<br>◯ susceptible |
| Amox/clav: Worst susceptibility from urine and blood collected during the first 24 hours of the first blood culture | ◯ resistant<br>◯ susceptible |
| Cefazolin: Worst susceptibility from urine and blood collected during the first 24 hours of the first blood culture | ◯ resistant<br>◯ susceptible |
| Cefurox: Worst susceptibility from urine and blood collected during the first 24 hours of the first blood culture | ◯ resistant<br>◯ susceptible |
| ***ceftriaxone: Worst susceptibility from urine and blood collected during the first 24 hours of the first blood culture*** | ◯ resistant<br>◯ susceptible |

| | |
|---|---|
| Cefepime: Worst susceptibility from urine and blood collected during the first 24 hours of the first blood culture | ◯ resistant<br>◯ susceptible |
| Cipro: Worst susceptibility from urine and blood collected during the first 24 hours of the first blood culture | ◯ resistant<br>◯ susceptible |
| Levo: Worst susceptibility from urine and blood collected during the first 24 hours of the first blood culture | ◯ resistant<br>◯ susceptible |
| FQ resistance? | ______________________ |
| Gentamicin: Worst susceptibility from urine and blood collected during the first 24 hours of the first blood culture | ◯ resistant<br>◯ susceptible |
| Tobramycin: Worst susceptibility from urine and blood collected during the first 24 hours of the first blood culture | ◯ resistant<br>◯ susceptible |
| Pip/tazo: Worst susceptibility from urine and blood collected during the first 24 hours of the first blood culture | ◯ resistant<br>◯ susceptible |
| Sulfamethoxazole/Trimethoprim: Worst susceptibility from urine and blood collected during the first 24 hours of the first blood culture | ◯ resistant<br>◯ susceptible |
| Meropenem: Worst susceptibility from urine and blood collected during the first 24 hours of the first blood culture | ◯ resistant<br>◯ susceptible |
| ESBL detected:<br><br>NOTE:<br>-If field is already filled in, no action needed. Move on to next field.<br>-If field is BLANK, please review the blood and urine culture results. Does it say "Extended spectrum beta-lactamase" or "ESBL" somewhere in the comments? If yes, enter YES. If no, enter NO. | ______________________ |

______________________

# Inpatient antibiotics

| | |
|---|---|
| Time of first ACTIVE IV antibiotic<br><br>**please double check if this pre-populated data is correct by double checking urine and blood culture susceptibilities against the administered abx in the MAR summary | ______________________ |
| Time (hours) from index blood culture to intravenous antibiotic start date | ______________________ |
| ***Check MAR Summary***<br>Was an effective IV antibiotic started within 12 hours of blood culture collection time?<br><br>NOTE: Both the blood AND urine culture organism must be SUSCEPTIBLE | ◯ Yes<br>◯ No |
| ***Check MAR Summary***<br>Was an effective IV antibiotic started within 24 hours of blood culture collection time?<br><br>NOTE: Both the blood AND urine culture organism must be SUSCEPTIBLE | ◯ Yes<br>◯ No |
| Which antibiotic was given within 12 hours of the positive blood culture?<br><br>*Usually pre-populated, but please double check the MAR summary. If this field is blank or if incorrect, please update as needed*<br>To count as empiric therapy, the drug had to be given within 12 hours before or after the positive blood culture | ☐ Aminoglycoside<br>☐ Cefepime<br>☐ Ceftriaxone or cefotaxime<br>☐ Pip/tazo<br>☐ Carbapenem (includes ertapenem)<br>☐ Ceftazidime<br>☐ Other beta lactam antibiotic<br>☐ Ciprofloxacin<br>☐ Levofloxacin<br>☐ Trimethoprim/sulfamethoxazole<br>☐ Nitrofurantoin<br>☐ Fosfomycin<br>☐ Aztreonam<br>☐ ampicillin |
| IV antibiotic last INPATIENT dose administered (If patient had IV antibiotics administered 72 hours after their last dose, it would be considered another episode of IV antibiotics)<br><br><br>**MAR summary: make sure this pre-populated number is correct based on IV antibiotic days for INPATIENT ONLY. It may auto-calculate to include outpatient days too. Double check: the last IV antibiotic dose should NOT be AFTER the date of discharge. If this needs to be corrected, the Days of Therapy field below will ALSO need to be corrected!** | ______________________ |

TOTAL INPATIENT IV antibiotic days
(difference between the first and last INPATIENT dose of IV antibiotics)

**MAR summary: make sure this pre-populated number is correct based on IV antibiotic days for INPATIENT ONLY (between dates of admission and discharge). It may auto-calculate to include outpatient days too - needs to be corrected**

Total APPROPRIATE days of INPATIENT IV antibiotics

**NOTE, this only includes administration of IV antibiotics to which the organism was SUSCEPTIBLE based on blood and urine culture results (e.g. exclude days of antibiotics given if RESISTANT)**

***1-4 days IV abx***

***1-7 days IV abx***

Total APPROPRIATE IV + ORAL INPATIENT antibiotic days

(was patient switched to appropriate PO abx while still INPATIENT)
**Again, need to double check this includes INPATIENT days only

Appropriate inpatient PO abx days

Ampicillin dot (DOT calculations by calendar day are pre-populated - no need to fill in)

Aminoglycoside DOT

Cefepime DOT

DOT Cefotaxime or Ceftriaxone

pip/tazo DOT

DOT ceftazidime

DOT carbapenem (includes ertapenem)

DOT other beta lactam

DOT Intravenous ciprofloxacin

DOT PO cipro

DOT IV levofloxacin

DOT PO levofloxacin

DOT trimethoprim sulfa

DOT nitrofurantoin

DOT Fosfomycin

DOT aztreonam

DOT cephalexin

DOT cefuroxime oral

DOT cefpodoxime

DOT cefixime

DOT cefdinir

DOT amoxicillin

DOT amox/clav

DOT cefazolin

DOT cefuroxime

DOT cephamycin

DOT ceftaroline

DOT Unasyn

DOT ceftaz/avi OR ceftolozane/tazo OR meropenem/vabor

DOT IV sulfatrim

# Discharge abx

| | |
|---|---|
| Days supply of the first outpatient antibiotic prescription generated on the day of discharge.<br><br>NOTE:<br>Please verify days supply in the Discharge Summary note. This pre-populated field is not always correct from EDW - please update if incorrect | __________________________ |
| **Calculate TOTAL APPROPRIATE antibiotic days for UTI/bacteremia including BOTH inpatient and outpatient ACTIVE antibiotics.<br><br>Please calculate this independent of redcap numbers | __________________________ |
| Estimated total inpt + outpt duration (based on manual Rx) | __________________________ |
| Estimated total inpt + outpt duration (based on electronic Rx) | __________________________ |
| IF IV antibiotics restarted after 72 hours without a dose of IV antibiotic, this is the duration of the second antibiotic course.<br>(leave blank if not pre-populated) | __________________________ |
| total number of discharge antibiotic prescriptions generated at discharge | __________________________ |
| Was at least one antibiotic prescribed for the treatment of UTI/bacteremia? | ◯ Yes<br>◯ No |
| Was an IV antibiotic dispensed at discharge? | ◯ Yes<br>◯ No |
| total appropriate inpatient + outpatient PO abx days | __________________________ |
| Discharge IV therapy. Please select discharge medications? | ☐ Ceftriaxone<br>☐ Ertapenem<br>☐ Meropenem<br>☐ Other |
| PICC line placed in central within 48 hrs of discharge? (based on charge code) | ◯ Yes<br>◯ No |

PICC line or other central line placed for outpatient IV antibiotics?

◯ Yes
◯ No

NOTES:
STEP 1. Review Discharge Summary Note (Use Control + F search function for "PICC" and "Midline"). If the Discharge note says one of these lines was placed, mark YES. Otherwise, proceed to STEP 2.

STEP 2. Review flowsheets for line documentation
1. Go to Results Review --> Vitals - Recent (Table view)
2. Change the flowsheet to LinesTubesDrains
3. Right click in grey bar to Change Search Criteria, change start/stop dates to 1 day before admit and 1 day after discharge
4. Click on Central Line in the Navigator pane
5. Review for any central lines placed (usually PICC - Peripherally Inserted Central Catheter, or Midline)
6. The Activity should say "Inserted" and the Current Indication should say something about long term or IV antibiotics. These are typically inserted within 48 hours of discharge.

---

Please select all ORAL antibiotics prescribed for the treatment of bacteremia at discharge

☐ Oral levofloxacin
☐ Oral Ciprofloxacin
☐ Oral cephalexin
☐ Oral cefuroxime
☐ Oral cefpodoxime
☐ Oral cefdinir
☐ Oral cefixime
☐ Oral Trimethoprim/sulfamethoxazole
☐ Oral nitrofurantoin
☐ Oral amoxicillin
☐ Oral amoxicillin/clavulanate
☐ Oral fosfomycin
☐ Other oral antibiotic

---

***Fluoroquinolone (referent group)*** ______________________________

***Trimethoprim-sulfamethoxazole*** ______________________________

***Highly Bioavailable Beta-Lactam*** ______________________________

***Low Bioavailability Beta-Lactam*** ______________________________

***ANY Beta-Lactam*** ______________________________

Was at least one discharge prescription given with activity against blood and urine culture organism?

NOTE
-Check susceptibilities against discharge prescription
-If patient discharged with an INACTIVE antibiotic (i.e. antibiotic to which the blood or urine culture is resistant), please enter a comment "ID Pharmacist to review"

◯ Yes
◯ No

**Leave blank, ID Pharmacist to fill in**
Was a change made to a new ACTIVE antibiotic following discharge?

◯ Yes
◯ No

How long (in days) did it take for the new ACTIVE antibiotic to be prescribed? ______________________

New ACTIVE antibiotic called in (abx name, dose, route, duration) ______________________

First antibiotic name at discharge ______________________

First abx dose ______________________

First abx route ______________________

First abx frequency ______________________

Days supply of discharge antibiotic (calculated) ______________________

First abx sig ______________________

Second antibiotic name at discharge ______________________

Second abx dose ______________________

Second abx route ______________________

Second abx frequency ______________________

Days supply drug 2 ______________________

Second abx sig ______________________

| | |
|---|---|
| Third antibiotic name at discharge | __________________________________ |
| Third abx dose | __________________________________ |
| Third abx route | __________________________________ |
| Third abx frequency | __________________________________ |
| Days supply third drug (calculated) | __________________________________ |
| Third abx sig | __________________________________ |
| The lowest CrCl within 24 hours of discharge was: [crcldsch] mL/min<br><br>Was the DISCHARGE antibiotic dose appropriate for renal function?<br><br>NOTE - use Intermountain Renal Dosing Guideline as a reference!! Look for recommended dosing for bacteremia or severe infections.<br>-Cephalexin 500mg or 1g q6h is ok | ◯ appropriate<br>◯ too low |
| Cation or Multivitamin included in H&P or d/c summary?<br><br>Iron, Mg, Calcium, Aluminum, Sucralfate, or Multivitamin containing any of the above | ◯ Yes<br>◯ No |

# Readmission evaluation

| | |
|---|---|
| Death date (within 90 days of bacteremia)<br><br>*pre-populated, no action needed if blank* | __________________ |
| ***CENSORING***<br>Time to Death (days) | __________________ |
| Mortality attributed to initial UTI treatment?<br>Defined as ANY of the following within 90 days:<br>-Positive blood culture (same organism) at time of death/withdrawal of care<br>-Positive urine culture (same organism) + sepsis at time of death/withdrawal of care<br>-Death due to C.diff<br><br>**if cause of death is unknown, then mark No | ◯ Yes<br>◯ No |
| C diff test within 90 days of first positive blood culture<br><br>(Pre-populated. No action needed if blank) | ◯ Yes<br>◯ No |
| Clostridium difficile positive, defined as either toxin or PCR for toxin positive any method (within 90 days)<br><br>(Pre-populated. No action needed if blank) | ◯ Yes<br>◯ No |
| Date of positive C.diff test | __________________ |
| Time to positive C.diff test (days from discharge) | __________________ |
| Date of FIRST readmission to hospital within 90 days<br><br>*Validate manually if no date is pre-populated* | __________________ |
| Time to hospital readmission | __________________ |
| Date of FIRST ED visit within 90 days<br><br>*Enter manually* | __________________ |
| Time to ED readmission | __________________ |
| Date of FIRST outpatient abx given within 7-90 days of discharge<br><br>*Enter manually* | __________________ |
| Time to first outpatient abx between 4-90 days | __________________ |

| | |
|---|---|
| Was there an all-cause UNPLANNED readmission to the ED or hospital within 90 days? | ◯ Yes<br>◯ No |
| Was the UNPLANNED readmission related to a UTI?<br><br>Notes:<br>-Read the ED note and/or H&P: was UTI listed as one of the primary diagnoses/reasons for admission? If so, mark YES. If not, mark NO | ◯ Yes<br>◯ No |
| Were ANY antibiotics given during the unplanned ED/hospital readmission? | ◯ Yes<br>◯ No |
| ***CENSORING***<br>Time to UTI-related readmission --OR--<br>Time to admission during which abx were given (days) | ______________________ |
| ***CENSORING***<br>Did patient have a follow-up healthcare encounter more than 90 days AFTER the index bacteremia? | ◯ Yes<br>◯ No |
| ***CENSORING***<br>Date of LAST KNOWN follow-up encounter within 90 days | ______________________ |
| Time to LAST KNOWN follow-up encounter within 90 days | ______________________ |
| Case notes/summary:<br>Write any notes about the case here (if needed) to help to clarify collected data<br><br>**Please write a brief note explaining any readmissions, recurrences, or mortality if applicable** | ______________________<br>(Example 1: Possible cholecystitis ruled out by surgery consult + imaging; repeat ED visit for unrelated chest pain. Example 2: Patient was lost to follow-up. Last known healthcare encounter was the date of discharge. ) |
